# Pathways to the Brain: Impact of Fine Particulate Matter Components on the Central Nervous System

**DOI:** 10.3390/antiox14060730

**Published:** 2025-06-14

**Authors:** Yasuhiro Ishihara, Miki Tanaka, Naoyuki Nezu, Nami Ishihara, Ami Oguro, Christoph F. A. Vogel

**Affiliations:** 1 Program of Biomedical Science, Graduate School of Integrated Sciences for Life, Hiroshima University, Hiroshima 739-8521, Japan; 2 Graduate School of Biomedical and Health Sciences, Hiroshima University, Hiroshima 734-8553, Japan; aoguro@hiroshima-u.ac.jp; 3 Department of Environmental Toxicology, University of California, Davis, CA 95616, USA; cfvogel@ucdavis.edu; 4 Center for Health and the Environment, University of California, Davis, CA 95616, USA

**Keywords:** PM_2.5_, components, brain, oxidative stress, inflammation, neurological disorders

## Abstract

Fine particulate matter with an aerodynamic diameter ≤ 2.5 µm (PM_2.5_) has been extensively studied due to its adverse health effects. Most research has focused on its impact on the respiratory system; however, increasing attention is being directed toward its effects on the brain. Associations between air pollution and neurological disorders—such as Alzheimer’s disease, cerebral infarction, and autism spectrum disorder—have been reported, with mechanism-based studies in animal models providing further insights. PM_2.5_ comprises a complex mixture of thousands of chemical constituents. To elucidate its neurotoxicity mechanisms, it is essential to investigate both its transport pathways to the brain and the specific actions of its individual components. This review highlights key PM_2.5_ components—water-soluble ions, metals, carbonaceous particles, polycyclic aromatic hydrocarbons, quinones, plastics, and bioaerosols—and outlines their potential routes of entry into the central nervous system, along with their associated mechanisms of action. By integrating these findings, this review contributes to a deeper understanding of the neurological effects mediated by PM_2.5_, which represent one of the most critical aspects of its health impact.

## 1. Introduction

Following the Industrial Revolution, air pollution became increasingly prevalent due to rapid technological advancements and expansion of factory-based industries. One of the notable events that highlighted the harmful effects of air pollution on human health was the Great Smog of London in 1952. This event, caused by accumulation of a strong acidic fog from combustion-related emissions, resulted in significant increases in respiratory illness and fatalities. In Japan, from the late 1950s to the 1970s, a similar public health crisis occurred in Yokkaichi, Mie Prefecture, where a high prevalence of asthma cases was linked to inhalation of air polluted with a large amount of sulfur oxides (SOx) produced by industrial combustion processes. These events prompted extensive research into the health impacts of air pollution.

The study of respirable particles has evolved considerably over the years, transitioning from a focus on total suspended particles (TSPs) to more refined categories such as particulate matter with an aerodynamic diameter ≤ 2.5 µm (PM_2.5_), and ultimately to nanoparticles. TSPs include all airborne particles, regardless of size, and have historically been the primary focus of air quality studies. TSP exposure is linked to respiratory problems, including chronic cough, bronchitis, and chest illnesses, particularly in children [1]. However, its broad categorization has limited the understanding of specific health impacts. Particulate matter with an aerodynamic diameter ≤ 10 µm (PM_10_) is more relevant for health impact studies as it can penetrate deep into the lungs, whereas TSPs encompass a broader range of particle sizes, making PM_10_ a more specific and critical focus for health-related research [2]. The “Harvard Six Cities Study” established the relationship between PM_2.5_ and adverse health effects using a dichotomous sampler with 15 and 2.5 µm 50% cut-points on its inlets for total and fine particle size fractions, respectively [3,4,5,6]. According to this study, the relative risk increase per 25 µg/m^3^ PM_2.5_ concentration was 1.36-times (95% confidence interval [CI]: 1.11, 1.68) for all deaths, 1.51-times (95% CI: 0.75, 3.09) for lung cancer deaths, 1.51-times (95% CI: 1.16, 2.00) for cardiopulmonary disease deaths, and 1.01-times (95% CI: 0.73, 1.42) for deaths due to other causes, indicating the risk of PM_2.5_ to respiratory and circulatory systems. Subsequent epidemiological studies have reinforced these findings. For example, a 10 µg/m^3^ increase in PM_2.5_ concentration has been associated with a 2.07% increase in hospitalization rates due to respiratory disease [7], and a 1.28% increase in the risk of heart failure [8]. Particle size is considered to be fundamental for entrance and deposition in the respiratory system. PM_10_ can penetrate the upper respiratory tract and reach the airways; PM_2.5_ can penetrate deeper into the respiratory system, reaching the tracheobronchial and pulmonary regions; and particulate matter with an aerodynamic diameter ≤ 1 µm (PM_1_) can reach the deepest parts of the lungs, including the alveolar region, where they can enter the bloodstream. Particles in the bloodstream trigger a cascade of toxic biological processes including oxidative stress, systemic inflammation, and endothelial dysfunction, which collectively exacerbate cardiovascular conditions.

In recent years, accumulating evidence has demonstrated that a portion of PM_2.5_ can reach the brain and impact the central nervous system (CNS) function. Epidemiological studies, in particular, have highlighted a link between air pollution and neurological disorders. For example, in China, each 10 µg/m^3^ increase in PM_2.5_ is associated with an increase in hospitalizations due to acute cerebrovascular events and transient stroke [9]. Additionally, exposure to PM_2.5_ has been linked to increased risk of Alzheimer’s disease (AD) [10]. Therefore, investigating the effects of PM2.5 on the CNS is important for explaining the overall health impact of PM_2.5_. Interpreting the effects of PM_2.5_ on the brain is complex, because the brain is relatively far from the external environment compared to the respiratory and circulatory systems. Therefore, it is essential to identify the specific components of PM_2.5_ that affect the brain and clarify the routes through which they exert their influence. In this review, we summarize the major components of PM_2.5_, discuss the pathways through which these components affect the brain, and mention their actions in the brain.

## 2. Components of PM_2.5_ and Their Biological Effects

**Figure 1:**
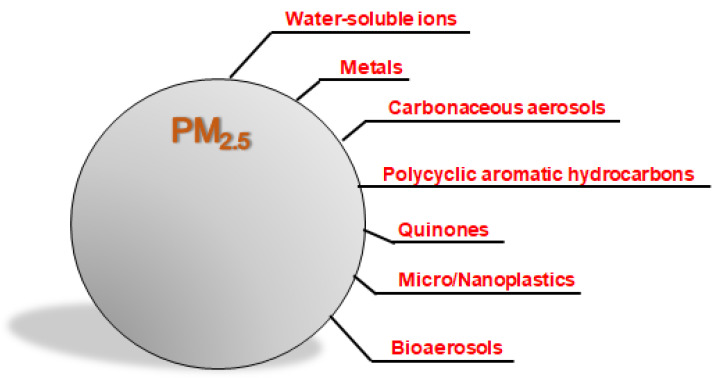
Components of PM_2.5_. PM2.5 is a complex mixture of various chemical components, reflecting its diverse sources and formation processes. The chemical composition of PM2.5 includes water-soluble ions, metals, carbonaceous aerosols, polycyclic aromatic hydrocarbons, quinones, plastics, and bioaerosols.

Understanding the specific components of PM_2.5_ is critical for elucidating its impact on human health at the molecular level. PM_2.5_ comprises a complex mixture of substances, including water-soluble ions, metals, carbonaceous aerosols, organic compounds, plastics, and bioaerosols (Figure 1), reflecting its diverse sources and formation processes.

### 2.1. Water-Soluble Ions

Water-soluble ions in PM_2.5_ primarily include sulfate ions (SO_4_^2−^), nitrate ions (NO_3_^−^), and ammonium ions (NH_4_^+^), which are produced when gaseous emissions from vehicles and industrial activities undergo atmospheric reactions. Although relatively non-toxic in small quantities, these deliquescent ions absorb moisture from the atmosphere. However, due to the limited availability of atmospheric moisture, the presence of sulfate and nitrate ions in PM_2.5_ results in a highly acidic particle surface. When acidic PM_2.5_ is deposited in the airways and lungs, it lowers tissues pH, triggering oxidative stress and inflammation. Notably, increases in atmospheric concentrations of sulfate and nitrate ions have been associated with respiratory system inflammation and subsequent bronchiolitis [11,12].

### 2.2. Metals

PM_2.5_ contains various metals, many of which are classified as heavy metals and exhibit toxicity even at low concentrations. According to the Ministry of the Environment in Japan, metals recommended for measurement when analyzing PM_2.5_ components include sodium (Na), aluminum (Al), potassium (K), calcium (Ca), scandium (Sc), vanadium (V), chromium (Cr), iron (Fe), nickel (Ni), zinc (Zn), arsenic (As), antimony (Sb), and lead (Pb). Specific metals can be traced to particular sources; therefore, measuring their concentrations in PM_2.5_ helps identify emission origins. For example, Fe is typically derived from soil, Pb from coal combustion, Cd from coal combustion and batteries (batteries are often incinerated as waste), Zn from tires and brakes, As from coal used in thermal power generation, V and Ni from petroleum combustion in thermal power generation, and Na from seawater [13,14,15]. In addition, manganese (Mn) in PM_2.5_ is primarily sourced from industrial activities, mining operations, and vehicular emissions. Mn exposure through PM_2.5_ is associated with adverse health effects, particularly affecting motor and cognitive functions. The US EPA has established reference concentrations to mitigate these risks [16].

The primary toxicological mechanism of these metals involves induction of oxidative stress via increased production of reactive oxygen species (ROS). The mechanism by which metals produce ROS involves several pathways that are primarily driven by the redox activity of metal ions. These pathways include direct electron transfer reactions such as the Fenton and Haber–Weiss reactions. In the Fenton reaction, redox-active metals such as Fe and copper (Cu) catalyze the conversion of hydrogen peroxide (H_2_O_2_) into highly reactive hydroxyl radicals (•OH). This process is a significant source of ROS in biological systems and is facilitated by cycling of metal ions between different oxidation states [17]. The Haber–Weiss reaction involves the interaction of superoxide anions (O_2_^•−^) with H_2_O_2_, catalyzed by metal ions, resulting in •OH production. The Haber–Weiss reaction is often coupled with the Fenton reaction, thereby enhancing overall ROS generation. Redox-active metals also interact with amyloid-beta peptides, leading to ROS production and contributing to oxidative stress and neurodegeneration [18]. The redox interactions between metals and amyloid-beta are thought to exacerbate the pathological processes in AD, highlighting the potential for metal-targeted therapies. Although less common, Mn and Ni can participate in O_2_ activation and ROS generation, particularly in specific enzymes and synthetic complexes [19].

In addition to redox reactions, metal and metal oxide nanoparticles can generate ROS through corrosion processes and surface defects. These defects facilitate electron transfer, leading to ROS formation. Surface properties of nanoparticles, including charge and composition, significantly influence their ROS-generating capabilities [20]. Furthermore, transition metals can enhance ROS production through photo-oxidation processes, wherein light energy promotes electron transfer, enhancing ROS production—particularly under atmospheric conditions involving interactions with organic compounds. Conversely, redox-inactive metals, such as Pb, Cd, and mercury, do not directly generate ROS; instead, they disrupt cellular antioxidant defense systems by depleting thiol-containing antioxidants and enzymes such as glutathione (GSH) and catalase (CAT), thereby reducing the cellular ability to neutralize ROS [21].

### 2.3. Carbonaceous Aerosols

Carbonaceous aerosols, which are produced by incomplete combustion of fossil fuels and biomass, account for approximately one-third of total PM_2.5_. These aerosols are typically categorized into two major components: elemental carbon (EC) and organic carbon. EC—also known as black carbon (BC)—is primarily emitted from vehicle exhaust. In urban areas of China, the mass concentration of BC ranges from 1.0–7.5 µg/m^3^ [22], which is significantly higher than levels observed in many developed countries [23]. Similarly, the majority of PM_2.5_ in India is predominantly composed of carbonaceous aerosols, underscoring their significance in evaluating regional air quality and associated health impacts [24].

Among carbonaceous components, BC has been relatively well studied in terms of its health effects. BC exerts toxicity primarily through oxidative stress and inflammation [25,26]. Due to its large surface area, BC adsorbs a wide range of organic and inorganic substances, including polycyclic aromatic hydrocarbons (PAHs) and toxic metals. The interaction between BC and heavy metals—such as Cu, Ni, Cd, Pb, and Fe—both in vitro and in vivo, shows synergistic toxic effects in alveolar epithelial cells, which leads to increased oxidative stress and cell death [27]. The physicochemical properties of BC—such as particle size, surface area, and surface charge—vary depending on the emission source, influencing its in vitro toxicity. Emissions from solid fuel combustion contain higher BC levels than those from motor vehicles [28]. Li et al. reported that BC from diesel exhaust exhibits the highest oxidizing potential among BC derived from three major emission sources in China (residential coal combustion, biomass burning, and diesel engine exhaust), as measured by a dithiothreitol assay [29].

BC can also promote ROS generation through redox oscillations, particularly in environments such as wetlands and farmlands where conditions alternate between microbial reduction and atmospheric oxidation. This cycling process significantly enhances ROS production, contributing to oxidative stress in these environments [30]. In human alveolar epithelial cells, exposure to BC nanoparticles has been shown to elevate ROS levels and disrupt Ca homeostasis. This oxidative stress is associated with mitochondrial dysfunction and is regulated by Ca-binding proteins such as calmodulin [31]. Although the mechanisms underlying BC-induced oxidative stress are well-established, it is essential to consider its broader environmental and health implications. Interactions between BC and other environmental pollutants, such as ozone, may alter its toxicological profile, potentially enhancing or attenuating its oxidative stress potential [32]. Furthermore, the role of antioxidants and potential protective interventions for mitigating BC-induced oxidative stress remains an important area of ongoing research.

### 2.4. PAHs

The organic fraction of PM_2.5_ is highly diverse, comprising approximately 10^4^ different organic compounds released into the atmosphere [33]. Among these organic components, PAHs have been extensively studied and have attracted considerable attention. PAHs form chemical bonds with DNA, leading to mutations and carcinogenesis. This process involves metabolic activation of PAHs into reactive intermediates (e.g., diol epoxides) which bind to DNA and cause genotoxic effects [34]. PAHs, such as benzo[a]pyrene and benzo[k]fluoranthene, are known to increase intracellular ROS levels, which is primarily attributed to their metabolic activation into reactive intermediates that directly generate ROS [35,36]. For example, benzo[a]pyrene induces oxidative stress by enhancing ROS production and disrupting antioxidant defense mechanisms. This disruption involves both a reduction in the levels of non-enzymatic antioxidants such as GSH, and altered activity of antioxidant enzymes, including superoxide dismutase and CAT.

PAHs also act as ligands for the aryl hydrocarbon receptor (AhR). AhR is a cytoplasmic transcription factor associated with several chaperone proteins, including HSP90. Upon ligand binding, the AhR complex translocates to the nucleus, where it forms a heterodimer with the AhR nuclear translocator and binds to dioxin/xenobiotic response elements in the promoter regions of target genes, thereby initiating transcription [37]. AhR activation induces expression of cytochrome P450 enzymes such as CYP1A1 and CYP1B1, which are involved in metabolism of xenobiotics and endogenous compounds. These enzymatic reactions generate ROS as by-products, further contributing to oxidative stress [35]. Exposure to AhR ligands significantly upregulated expression of the NADPH oxidase subunit p47phox, leading to NADPH oxidase activation and subsequent superoxide generation [38].

AhR is closely associated with inflammatory responses. Cyclooxygenase-2 (COX-2) contains a dioxin response element in its promoter region, and polychlorinated dibenzodioxins such as 2,3,7,8-tetrachlorodibenzo-p-dioxin (TCDD) induce COX-2 expression and prostaglandin synthesis [39]. COX-2 expression was upregulated in a dose-dependent manner in human macrophages after exposure to air pollution particles [40]. Expression of other inflammatory molecules, such as interleukin-1β (IL-1β) [41], interleukin-6 [42], interleukin-8 [43], interleukin-17 [44], interleukin-22 [45], interleukin-33 (IL-33) [37], and tumor necrosis factor α [46], was also upregulated by AhR. PAHs, including those present in PM_2.5_, significantly elevated IL-33 expression in macrophages [47]. Thus, PAHs can induce inflammatory reaction via AhR.

### 2.5. Quinones

Quinones are formed in the atmosphere primarily through photooxidation of PAHs, which are abundant in urban environments due to vehicular emissions and industrial activities. For example, the common PAHs naphthalene and phenanthrene react with •OH to form quinones such as 1,2-naphthoquinone and 9,10-phenanthrenequinone, respectively [48]. These compounds can also be directly emitted into the atmosphere from sources such as diesel exhaust particles and other combustion processes [49]. Quinones exist in both gas and particle phases in the atmosphere. Studies in Southern California have shown that vapor-phase concentrations of quinones are generally higher than those in the particle phase, with concentrations ranging from 80 pg/m^3^ for anthraquinone to 1747 pg/m^3^ for 1,4-naphthoquinone [50].

Toxicity of quinones is induced by two principal mechanisms [51,52]. The first is arylation of nucleophiles among important biological constituents; for example, quinones react covalently with thiols (e.g., GSH or the cysteine residues of proteins) to form arylation products that eventually cause cellular damage. The second mechanism is oxidative stress via redox cycling. Quinones undergo one-electron reduction to yield semiquinone radicals during the first step of this process. The semiquinone radicals are then reoxidized and can enter redox cycles in cooperation with molecular oxygen to form superoxide anions. These are converted into ROS, such as H_2_O_2_, the •OH, and singlet oxygen, through various pathways within cells.

N-(1,3-dimethylbutyl)-N′-phenyl-p-phenylenediamine (6PPD) is a chemical widely used in the rubber industry as an antioxidant and antiozonant to extend the lifespan of rubber products, such as tires. 6PPD reacts with atmospheric oxidants, leading to formation of 6PPD-quinones (6PPDQ). This reaction is facilitated by the presence of oxygen and ozone, which are abundant in the atmosphere, particularly in urban areas where tire wear particles are prevalent [53]. 6PPDQ is generated from 6PPD through a two-step phenyl hydroxylation process catalyzed by cytochrome P450 enzymes. This biotransformation occurs in both aquatic and mammalian species, with distinct species-specific metabolic kinetics observed. For example, fish liver microsomes show slower biotransformation rates than mammalian liver microsomes, indicating potential vulnerability of aquatic species to 6PPDQ exposure [54]. 6PPDQ also induces oxidative stress by producing ROS, which leads to oxidative damage to DNA, proteins, and lipids, similar to other quinones.

### 2.6. Plastics

Growing evidence indicates that microplastics (MPs) and nanoplastics (NPs) are significant environmental pollutants, with their presence in the atmosphere posing potential risks to ecosystems and human health. These plastic particles originate either from degradation of larger plastic debris, or are intentionally manufactured for specific industrial applications. Atmospheric transport facilitates widespread distribution of MPs and NPs, even to remote and pristine regions, emphasizing the need to understand their sources, distribution, and health impacts.

MPs and NPs have been detected in both urban and remote areas, indicating their capacity for long-range transport via atmospheric currents [55,56]. Major sources include road-related emissions (e.g., tire and brake wear and mismanaged plastic waste), which significantly contribute to atmospheric MPs. In contrast, marine sources, once considered primary contributors, have been shown to emit lower quantities than previously estimated [57]. Indoor environments also represent significant exposure pathways, with MPs commonly found in indoor air and dust. Atmospheric deposition rates of MPs vary widely, with reported concentrations ranging from 0.0065–1583 particles/m^3^, depending on the environment [55]. Quantifying NPs remains challenging due to their small size and the lack of standardized measurement methods. In a study conducted in urban Shanghai, fine plastic particles (FPPs) were found to constitute approximately 13.2% of PM_2.5_, as determined using an advanced aerosol enrichment system combined with spectroscopic techniques to distinguish FPPs from other carbonaceous particles [58].

MPs and NPs have been shown to increase ROS production across various models, including cell lines and animal systems. Elevated ROS levels can damage cellular macromolecules—such as DNA, proteins, and lipids—resulting in cellular dysfunction and apoptosis [59,60]. Mitochondrial damage (i.e., a significant source of ROS) is commonly observed following MP and NP exposure, further exacerbating oxidative stress and contributing to cellular aging and senescence. Particle size plays a critical role in toxicity. Due to their smaller size and larger surface area, NPs can reduce antioxidant enzyme activity and enhance oxidative stress more significantly than larger MPs [61].

MPs and NPs can also serve as vectors for harmful chemicals, such as bisphenol A and phthalates, which are known endocrine disruptors. The high surface area of NPs facilitates sorption of organic pollutants and heavy metals, amplifying their toxicological impact [62]. Oxidative stress can lead to increased production of inflammatory mediators. For example, polystyrene NPs have been shown to activate the cGAS-STING pathway, leading to nuclear factor-kappa B (NF-κB) signaling and upregulation of pro-inflammatory mediators [63].

Recently, plastic aging has gained attention as an important factor affecting human health. Exposure to ultraviolet radiation induces photooxidative degradation of plastic, leading to free radical formation and increased oxidative stress. This process is characterized by cleavage of chemical bonds, producing smaller fragments and environmentally persistent free radicals [64]. Oxidative degradation of common plastics, such as polyethylene and polypropylene, leads to formation of functional groups such as ketones and acids, driven by environmental conditions. Moreover, photodegradation of polyethylene terephthalate (PET) generates terephthalic acid (i.e., a monomer of PET), which weakly induces inflammation in macrophages. Thus, monomer release following photodegradation may contribute to the toxicity of degraded PET [65].

### 2.7. Bioaerosols

Among the constituents of PM_2.5_, bioaerosols are gaining increasing attention due to their potential health impacts. Bioaerosol refers to airborne particles of biological origin, ranging in size from 0.001–100 µm. These include whole microorganisms—such as fungi, bacteria, and viruses—as well as microbial fragments and components, such as lipopolysaccharide (LPS). Several airborne microorganisms, including *Escherichia coli*, *Legionella pneumophila*, and *Mycobacterium tuberculosis*, are known pathogens [66].

Bioaerosols originate from various natural and anthropogenic sources [67,68]. Natural sources include soil and water bodies. When rain disturbs soil or agitates water surfaces in rivers, lakes, and oceans, resident microorganisms can become aerosolized. For example, rainfall can mobilize approximately 0.01% of soil bacteria into the atmosphere, i.e., an estimated 1.2 × 10^22^ to 8.5 × 10^23^ bacteria per year. Anthropogenic sources also contribute significantly to bioaerosol levels. The human body harbors approximately 10^12^–10^14^ microorganisms, which can be dispersed into the air by coughing, sneezing, and movement. Bioaerosol concentrations near livestock farms have been reported at 10^3^–10^5^ CFU/m^3^, whereas fungal aerosol levels at sewage treatment facilities range from 6.3 × 10^2^ to 3.9 × 10^3^ CFU/m^3^. Some bioaerosols dispersed in the air attach to PM_2.5_, with the extent of attachment increasing under weak wind and high humidity conditions.

Fungi and bacteria are engulfed and digested by phagocytes, such as macrophages, through a process known as phagocytosis. This process is closely associated with production of ROS, including superoxide, via activation of the NADPH oxidase complex. This activation involves translocation of cytosolic components—such as p47^phox^, p67^phox^, and Rac-GTP—to the phagosomal membrane, where they interact with cytochrome *b_558_* to initiate superoxide generation [69].

LPS, a polysaccharide component of the outer membrane of Gram-negative bacteria, exhibits strong pro-inflammatory activity. Airborne LPS concentrations can increase under specific environmental conditions; for example, elevated levels of 1498 EU/m^3^ were detected at a livestock farm in the Netherlands [70]. The LPS content in PM_2.5_ collected in Yokohama, Japan, was approximately 10 EU/mg PM_2.5_, which is a level sufficient to induce inflammatory responses in macrophages [47]. The primary receptor for LPS is Toll-like receptor 4 (TLR4), a member of the TLR family. TLR4 is expressed on the cell membrane and forms a complex with the MD-2-related lipid recognition protein. Upon LPS binding, the TLR4-MD2 complex initiates intracellular signaling cascades that activates transcription factors such as AP-1 and interferon regulatory factor 3, leading to production of inflammatory cytokines and interferons [71,72].

## 3. Routes of PM_2.5_ Components into the Brain

**Figure 2:**
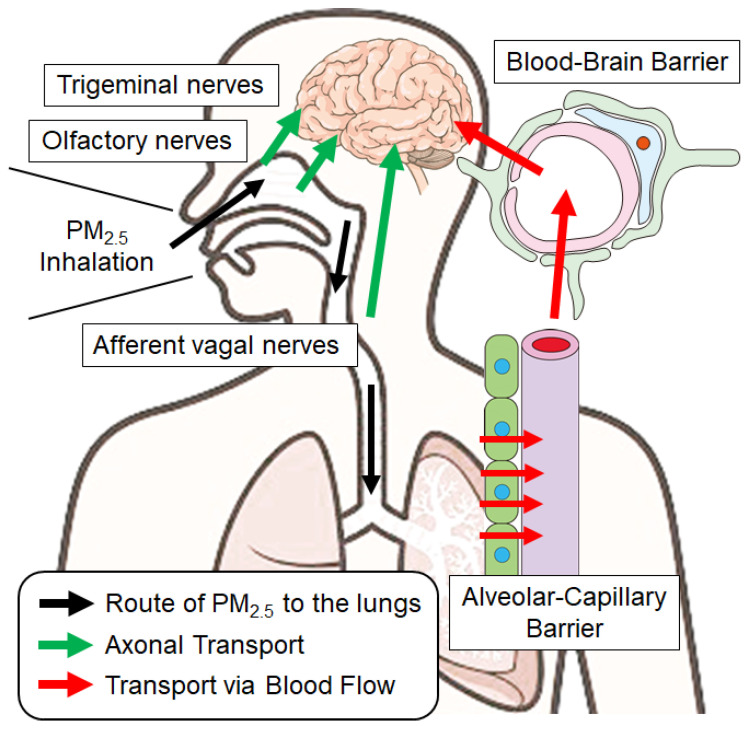
Translocation of inhaled solid PM_2.5_ particles into the brain. Inhaled solid PM_2.5_ particles are translocated to the brain by axonal transport using olfactory, trigeminal, and afferent vagal nerves. In another route, particles that reach the lungs can pass through the alveolar–capillary barrier, enter the blood, and cross the blood–brain barrier to reach the brain.

Inhaled PM_2.5_ can impact the brain through multiple pathways. Solid particles directly translocate to the brain (Figure 2), whereas chemical components dissolved in body fluids may exert indirect effects via peripheral mechanisms.

### 3.1. Solid Particles: Pathways via the Olfactory Epithelium

Understanding the routes and mechanisms by which PM_2.5_ and its components affect the brain is critical. PM_2.5_ primarily enters the body through inhalation, with the nose and mouth serving as the primary entry points. When nasally inhaled, a portion of PM_2.5_ is adsorbed in the nasal cavity, and the remainder reaches the lungs via the airways. Oral inhalation directly leads to deposition in the oral cavity and lungs. Oberdörster et al. demonstrated that exposure of rats to ^13^C-labeled fine particles via transient inhalation led to an immediate increase in the ^13^C signal in lungs. However, 1-week post-exposure, the ^13^C concentration in the lungs had decreased and was similar to that in the olfactory bulb [73]. Notably, the ^13^C concentration in the olfactory bulb increased 1-day post-exposure and remained stable for at least 1 week. These results suggest that PM_2.5_ can accumulate in the brain and that clearance from the brain is limited. Elder et al. investigated translocation of Mn nanoparticles to the CNS in rats. They compared animals with both nostrils patent to those with the right nostril occluded. In rats with patent nostrils, Mn accumulated in the striatum, frontal cortex, and cerebellum. In contrast, in rats with the right nostril occluded, Mn accumulation was limited to the left olfactory bulb. These findings indicate that the olfactory neuronal pathway is efficient for translocating inhaled solid nanoparticles to the CNS [74].

The olfactory epithelium is composed of neurons that detect odorant molecules. These olfactory neurons extend their axons to the surface of the olfactory epithelium through the cribriform plate—a porous bone in the nasal cavity—to transmit odorant signals to the olfactory bulb. It is hypothesized that inhaled nanosized particulate matter is taken up by the olfactory neurons and enters the olfactory bulb via axonal transport. Additionally, the nasal cavity is innervated by the maxillary branch of the trigeminal nerve, which projects to the spinal trigeminal nucleus. Lewis et al. demonstrated direct uptake and transport of inhaled MnCl_2_ aerosols in rats via the trigeminal system, reporting a small but significant increase in Mn levels in the spinal trigeminal nucleus [75]. These findings suggest that the trigeminal nerve can serve as an additional pathway for entry of inhaled Mn into the brain. In addition to the olfactory and trigeminal pathways, afferent vagal nerves innervating the airways are also considered a potential pathway for particle translocation to the brain [73]. Notably, >40 years ago, 0.5-μm polystyrene particles were observed moving through crab axons [76], demonstrating that exogenous particles can move within axons. Such particles may enter neurons via endocytosis and be expelled by exocytosis. Furthermore, magnetite particles have been detected in postmortem human brains using electron microscopy [77].

Generally, axonal transport is limited to particulate materials that are insoluble in biological fluids, such as metals, carbon, and plastics. Magnetite particles have also been detected in the human brain [77]. BC particles were directly visualized in the brains of four individuals with AD, even in deep regions such as the hippocampus and olfactory bulb [78]. MPs were also detected in the olfactory bulbs of eight out of fifteen individuals, with polypropylene being the most commonly identified polymer; MP sizes ranged from 5.5–26.4 µm, and the mean fiber length was 21.4 µm [79]. These findings support the hypothesis that insoluble particulate matter can translocate into biological fluids from the olfactory epithelium to the olfactory bulb. In addition, Garcia et al. developed an anatomically accurate model of the human nasal cavity based on computed tomography scans, and demonstrated that olfactory deposition of inhaled nanoparticles is highest for 1–2 nm particles with approximately 1% of inhaled particles depositing in the olfactory region; this deposition rate decreases sharply to 0.01% for particles with a 100 nm diameter [80]. However, a contradiction arises from the assumption that all transport of these particles in the brain exclusively occurs via axons. The diameter of the exon in a nerve cell is <1 µm, rendering transport of solid particles of submicron size through this pathway physically implausible. Therefore, although it is reasonable to infer that some of the solid particles present in the olfactory bulb may have reached the brain via axonal transport, the size constraints of axons suggest that alternative transport mechanisms must also be considered.

### 3.2. Solid Particles: Passing Through the Alveolar Barrier into the Blood

The alveolar barrier is a critical structure in the respiratory system, and is essential for maintaining lung function and integrity. It is primarily composed of two cell types: Type I and Type II pneumocytes. Type I cells constitute most of the alveolar surface and are primarily responsible for gas exchange, whereas Type II cells produce a surfactant which reduces surface tension and prevents alveolar collapse [81]. Capillaries lie beneath the alveolar–epithelial cells, supported by a thin basement membrane that facilitates efficient gas diffusion and prevents fluid accumulation [82].

The permeability of the alveolar barrier to fine particles is a key factor in understanding respiratory health impacts particularly in the context of air pollution and nanoparticle exposure. Fine particles can penetrate this alveolar–capillary barrier, potentially entering the systemic circulation and causing adverse health effects. Studies using in vivo and in vitro models have investigated the mechanisms and factors influencing this permeability. The structure of the alveolar–capillary membrane—including intercellular junctions and pinocytotic vesicles—plays a significant role. Among these, venular junctions are the most permeable [83]. Fine particles may be internalized by alveolar epithelial cells through endocytotic vesicles. This process allows particles to reach the basement membrane and potentially enter the bloodstream. Ultrafine particles have been detected in extrapulmonary organs shortly after inhalation, indicating their rapid translocation across the alveolar barrier [84]. Transcytosis is a cellular process that facilitates transport of macromolecules to traverse cells without passing through intercellular junctions. Transcytosis begins with endocytosis on one cell surface, followed by intracellular vesicle transport and exocytosis on the opposite cell surface [85]. In the alveolar epithelium, transcytosis plays a vital role in clearing inhaled nanoparticles such as silica. This process involves caveolin-mediated macropinocytosis and actin-dependent transport, contributing to lung homeostasis [86]. However, the factors determining the directionality of particle transcytosis remain unclear, although evidence supports their role in transporting particles from the alveolar space into the bloodstream. The ability of particles to cross the alveolar barrier is strongly influenced by their size and surface charge. Particles smaller than 16 nm can rapidly penetrate the barrier, whereas larger particles tend to remain trapped in the lungs. Neutrally charged particles also exhibit more rapid lung penetration than charged particles [87]. Additionally, ultrafine particles may cross cellular membranes through non-phagocytic mechanisms, such as passive diffusion or adhesive interactions. These mechanisms allow them to bypass traditional endocytic pathways and directly interact with intracellular components, potentially amplifying their toxicological impact [88].

Kidney biopsies from 25 transplant patients revealed BC particles, visualized using white light generation under femtosecond-pulsed illumination [89]. These particles were subsequently identified in the blood vessels and capillaries of 40% of the samples, indicating that BC particles can enter the blood. BC particles have also been detected in cord blood [90]. Additionally, Leslie et al. identified plastic particles ≥ 700 nm—such as polyethylene terephthalate, polyethylene, and polystyrene—in the blood of healthy human volunteers [91]. The mean total quantifiable concentration of plastic particles in the blood was 1.6 µg/mL. Particulates can cross the alveolar barrier, suggesting that some of the detected particles may have originated from inhaled sources. However, orally ingested particles can traverse the intestinal barrier and enter the blood [92], which complicates efforts to determine the source of particulates identified in human blood.

### 3.3. Solid Particles: Passing Through the Blood–Brain Barrier into the Brain

The blood–brain barrier (BBB) is a critical defense mechanism that protects the brain from harmful substances. It is composed of three cell layers: vascular endothelial cells, pericytes, and astrocytes [93]. Vascular endothelial cells form tight junctions that narrow the gap between cells, acting as a physical barrier. Pericytes surround these endothelial cells and contribute to vascular stability and regulation of blood flow. Astrocytes—a type of glial cell—reinforce the tight junctions, regulate vascular permeability, provide nutrients, and maintain brain homeostasis. Together, these components tightly regulate the passage of ions and other substances in the brain.

In addition to crossing the alveolar barrier, fine particles penetrate the BBB, and this ability is strongly influenced by their size. A study using an engineered BBB model found that smaller (0.2 µm) polystyrene microparticles exhibited significantly greater uptake and transendothelial transport than larger ones (1.0 µm) [94]. Furthermore, nanometer-sized particles (but not larger microparticles) could reach the brain shortly after administration, suggesting that only particles below a certain size threshold can effectively cross the BBB [95]. Fine particles are believed to cross the BBB through mechanisms similar to those used at the alveolar barrier, such as endocytosis and transcytosis.

Importantly, particles that enter the systemic circulation increase BBB permeability through neuroinflammation, decreased transendothelial electrical resistance, and disrupted tight junction proteins [96,97]. When the BBB is compromised, particles of a size that would not normally cross under physiological conditions may gain access to the brain. In such cases, the enhanced BBB permeability is a secondary effect of components of PM_2.5_. Oxidative stress and neuroinflammation have also been shown to contribute to BBB permeability [98,99]. Notably, particle migration into the brain can further increase BBB permeability, facilitating entry of additional particles and amplifying particle-induced neurotoxicity.

Emerging evidence indicates that the protein corona plays a critical role in transport of nanoparticles across the BBB. Upon exposure to physiological fluids, such as blood, nanoparticles rapidly adsorb a variety of biomolecules (predominantly proteins) onto their surface. The adsorbed layer—termed the protein corona—imparts a new biological identity to the nanoparticles and determines subsequent protein and cell interactions [100]. In addition to proteins, other biomolecules—such as lipids—have also been reported to adsorb onto nanoparticles [95]. Notably, cholesterol molecules in the corona may facilitate nanoparticle uptake across the BBB, whereas certain proteins may inhibit this process [95]. Further investigation is necessary to elucidate the role of the biomolecular corona in mediating nanoparticle transport across various biological barriers.

Considering the anatomical distances, the pathway from the nasal cavity to the olfactory bulb offers a more direct route to the brain, compared to the pathway from the alveoli, which must traverse both the alveoli–blood barrier and the BBB. Moreover, translocation of fine particles into the brain is influenced by various physicochemical characteristics, including particle size, surface charge, shape, and attached chemicals. Considering the diverse physical and chemical properties of environmental fine particles such as PM_2.5_, generalizing their brain migration behavior remains insufficient. Instead, understanding the translocation of such fine particles requires an integrated approach that considers both particle-specific properties and the physiological condition of the host.

### 3.4. Transport via Biological Fluids

Water-soluble ions rapidly dissolve upon contact with biological fluids. Due to their electric charge, they cannot passively diffuse through lipid membranes and require specific ion channels to pass through. Consequently, the primary target of water-soluble ions is the respiratory system, where they alter the pH and ionic strength. Historical events such as London smog demonstrated that inhalation of sulfurous smoky fog induced asthma, and in some cases, resulted in death [101]. Similarly, the Yokkaichi Asthma incident in Japan was linked to sulfur dioxide exposure [102]. Acidic vapors activated the capsaicin-sensitive sensory nerves in the airways, triggering inflammation and dysfunction. Mediators such as kinins, nitric oxide, ROS, and proteases contributed to bronchoconstriction and airway hyperreactivity [103]. Acidic environments also stimulate proton-sensing receptors, such as ovarian cancer G protein-coupled receptor 1, in airway smooth muscle cells. This stimulation leads to increased production of inflammatory cytokines (e.g., interleukin-6) and Ca^2+^ mobilization, both of which are involved in the inflammation and bronchoconstriction [104]. Although ion imbalance and pH disturbances can affect the brain, the quantity of inhaled water-soluble ions is insufficient to alter the ion balance or liquid properties of body fluids. Therefore, their direct impact on the brain is likely to be minimal.

PAHs are highly lipophilic, enabling them to readily cross cell membranes and distribute to various tissues. Following oral administration of ^3^H-labeled dibenzo[def,p]chrysene to female mice, high concentrations were initially detected in the stomach and intestines at 24 h; however, these declined after 1 week, and showed increased accumulation in the mammary gland and liver [105]. Additionally, orally administered PAHs are also transferred to the brain, although their amount varies depending on the type of PAHs [106]. Although experimental data on direct inhalation of PAHs are limited, it is assumed that PAHs inhaled with PM_2.5_ diffuse to various tissues and organs in the body, including the brain, making it a potential direct target. AhR is expressed in the brain [107,108]; therefore, PAHs that reach the brain may exert AhR-mediated effects. The ligand-activated AhR-mediated induction of biological responses by PAHs has been studied using chemically activated luciferase gene expression assays [109,110]. Compounds such as benzo[b]fluoranthene and benzo[k]fluoranthene strongly activate AhR [110]. Activation of the PAHs–AhR pathway can induce oxidative stress and inflammation. Benzo[a]pyrene-DNA adducts have been detected in brains of mice exposed to benzo[a]pyrene [111], with these mice also showing increased expression of the N-methyl-D-aspartate receptor subunits *Grina* and *Grin2a*. The N-methyl-D-aspartate receptor can induce excitotoxicity and subsequent oxidative stress via excess Ca^2+^ flux [112]; therefore, epigenetic mechanisms may contribute to PAH-induced neurotoxicity.

The ability of quinone compounds to penetrate into the brain is believed to depend on their structure, and it has been shown that at least some quinone compounds pass through the BBB and reach the brain. Ubiquinone (coenzyme Q10), a type of quinone, has been shown to cross the BBB when administered intravenously [113]. Morphine-2,3-quinone, a morphine metabolite, have been detected in the brain, indicating that it can cross the BBB under certain condition [114]. Although direct migration of 6PPDQ to the brain is not explicitly documented, the disruption of the BBB observed in the study could allow for the compound or its effects to reach the brain [115]. Therefore, oxidative stress induced by quinone compounds may play a fundamental role in the action of PM_2.5_ in the brain.

Live bacteria are phagocytosed and eliminated by macrophages. Alveolar macrophages—located in the alveolar cavity—phagocytose foreign substances and direct them towards lymphatic vessels for degradation and removal [116]. LPS—a component of the outer membrane of Gram-negative bacteria—is a glycolipid composed of a polysaccharide chain bound to a lipid moiety known as Lipid A. The Lipid A region is primarily responsible for the physiological activity of LPS, and can mediate physiological actions [117]. Due to its water solubility and high molecular weight, LPS cannot readily cross the alveolar–capillary barriers or the BBB. Therefore, LPS inhaled as part of bioaerosols is unlikely to reach the brain.

### 3.5. Alternative Pathways of PM_2.5_ Neurotoxicity

Recently, transient receptor potential channels (TRPCs) have attracted attention as potential targets of PM_2.5_. TRPCs are highly expressed in epithelial cells and exhibit sensory innervation at primary entry sites of the body, such as the skin and airways. Notably, the activation of transient receptor potential melastatin 2 by ROS leads to redistribution and degradation of the tight junction protein zona occludens-1, resulting in endothelial barrier disruption. This disruption facilitates gap formation, and subsequently leads to vascular hyperpermeability [118]. Similarly, activation of the transient receptor potential vanilloid 1 channel promotes airway vascular permeability [119]. Therefore, TRPCs may indirectly contribute to PM_2.5_-induced effects in the brain. In addition to its effect on TRPCs, PM_2.5_ damages the blood–retinal barrier, potentially allowing it to enter the circulatory system through this alternate route [120]. Moreover, PM_2.5_ exposure disrupts not only pulmonary function but also alters the gut microbiota and its metabolic profile [121,122]. The gut microbiome plays a critical role in synthesis of neurotransmitters such as dopamine and gamma-aminobutyric acid, and in modulating brain cytokine production [123,124,125]. Multiple pathways connect peripheral tissues to the brain, and these pathways may interact in complex ways to mediate the neurological effects of PM_2.5_ exposure.

## 4. Action of PM_2.5_ Components in the Brain

### 4.1. Action Mechanisms of Particles Delivered to the Brain

The complexity of the brain arises from its vast population of neurons and their intricate interconnections, which enable the sophisticated processing and integration of information. The brain networks exhibit remarkable plasticity, with synaptic connections being modifiable based on prior activity—an essential feature underlying learning and memory [126]. In addition to neurons, glial cells play crucial roles in maintaining and supporting the neuronal microenvironment. There are three primary types of glial cells: astrocytes, oligodendrocytes, and microglia [127]. Astrocytes regulate homeostasis, contribute to formation of the BBB, and modulate neurotransmitter levels. Microglia serve as the brain resident immune cells, performing immune surveillance and responding to injury through CNS remodeling. Oligodendrocytes produce myelin, a lipid-rich sheath that insulates axons and facilitates rapid electrical signal transmission.

Exposure to mine-site-derived particulate matter increased the protein expression of the ionized Ca binding adaptor molecule-1 (a marker of activated microglia) and the glial fibrillary acidic protein (a marker of astroglial injury) in the right hemisphere of the mouse brain, including the cortex and hippocampus [128]. These changes were accompanied by reduced exploratory behavior and potential memory impairment, suggesting that metal-containing particles could affect neurons, astrocytes, and microglia. Among the three cell types, microglia have been most studied in terms of their interaction with particles. Microglia exhibit high phagocytic activity and can phagocytose particles that have invaded the brain. Upon activation, they also cause oxidative stress [129]; for example, microglia exposed to silica nanoparticles show increased intracellular ROS production [130]. Similarly, polystyrene MPs administered to mice were found to accumulate in microglia, inducing neuroinflammation characterized by of NF-κB activation and elevated levels of pro-inflammatory cytokines [131]. These findings suggest that microglia are primary targets of solid particles that penetrate the brain. Following phagocytosis, microglia mediate oxidative stress and initiate inflammatory responses, such as upregulation of cytokines. The TLR4/NF-κB signaling pathway plays a crucial role in PM_2.5_-induced microglial activation and inflammation [132]. PM_2.5_ exposure also activates the NLR family pyrin domain-containing 3 (NLRP3) inflammasome, leading to increased IL-1β production and exacerbation of inflammatory processes [133]. The interplay between oxidative stress and TLR4/NF-κB signaling amplifies neuroinflammation, contributing to neuronal damage and neurodegeneration [132]. In AD models, PM_2.5_ exposure aggravates amyloid beta-induced neuronal injury via ROS generation and NLRP3 inflammasome activation [133]. The resulting microglial inflammation impairs neural function and promotes neurodegeneration [134]. Notably, the effects of PM_2.5_ on microglia, including oxidative stress and inflammation, are critical factors in the development and exacerbation of CNS demyelinating disorders [135]. The interaction between microglia and oligodendrocytes under PM_2.5_ exposure presents a promising avenue for future research.

Exposure to PM_2.5_ during critical developmental periods leads to autism spectrum disorder (ASD)-like features in animal models, with astrocyte activation being a contributing factor [136]. In cultured astrocytes, PM_2.5_ exposure induced both A1 and A2 phenotypes, characterized by increased expression of genes such as *Fkbp5*, *Sphk1*, *S100a10*, and *Il6*. Notably, A2-type activation leads to upregulation of neurotrophic factors, including Gdnf and Ngf, which are crucial for neuronal survival and function [137]. These findings suggest that PM_2.5_-induced astrocyte activation may exert both neuroinflammatory and neuroprotective effects.

Heavy metals exhibit strong binding affinities for several functional proteins in the brain, including N-methyl-D-aspartate (NMDA) receptors, Na^+^/K^+^-ATPase, and Ca^2+^ transporters [138]. Ions such as Pb^2+^, Ni^2+^, and Mn^2+^ can bind to NMDA receptors, enhancing glutamate uptake, which leads to excitotoxicity, oxidative stress, and subsequent cellular damage, Additionally, Zn^2+^, Ni^2+^, Pb^2+^, Cu^2+^, and Mn^2+^ exhibit an affinity for the MK-801 site, and inhibit the activity of NMDA receptors [139]. Inhalation of Pb and Cd activates voltage-dependent Ca channels [140]. Heavy metal exposure during the developmental stage disrupts mitochondrial bioenergetics and glycolysis, contributing to the pathophysiology of ASD [141]. Collectively, metal particles delivered to the brain may induce oxidative stress through both ion channel- and mitochondria-dependent mechanisms, resulting in neuronal injury.

MPs and NPs can induce oxidative stress, inflammation, and mitochondrial dysfunction—key processes underlying neurotoxicity. These processes can lead to neuronal apoptosis, neuroinflammation, and disruption of neurotransmitter systems, contributing to cognitive impairments and neurodegenerative changes [142]. Notably, photoaged MPs, such as polystyrene, exhibit greater neurotoxic potential than their virgin forms, affecting neurotransmitter levels and expression of neurotransmission-related genes. This disruption results in abnormal neurotransmission and neurodegeneration [143]. Although current evidence underscores the neurotoxic risks associated with MPs and NPs, considering these findings within the broader context of environmental pollution and its cumulative impact on human health is crucial.

In addition to the effects described above, various other components of PM_2.5_ exert distinct effects, as summarized in Table 1. Under physiological conditions, bioaerosols do not reach the CNS; therefore, only their role in BBB disruption is included in Table 1.

### 4.2. Indirect Effects of PM_2.5_-Induced Peripheral Oxidative Stress and Inflammation

Inhalation of PM_2.5_ induces oxidative stress and inflammation in peripheral tissues, particularly in the lungs [169,170,171]. Systemic oxidative stress can significantly impact the brain, as demonstrated in neurodegenerative diseases where systemic markers of oxidative stress correlate with brain damage [172]. Oxidative stress can compromise the integrity of the BBB, facilitating entry of harmful substances to the brain and exacerbating oxidative damage. This BBB disruption is a common pathological feature of neurodegenerative diseases and contributes to further neuronal damage [173]. Sustained PM_2.5_ exposure induces cardiac oxidative stress and dysfunction, which may worsen outcomes following ischemic stroke in mice [174]. Thus, oxidative cardiovascular damage linked to PM_2.5_ can affect cerebral circulation and stroke prognosis. Exposure to fine particles increases BBB permeability; for example, apolipoprotein E knockout mice exposed to mixed vehicle exhaust for 30 d showed reduced expression of the tight junction protein claudin-5 and increased levels of the inflammatory cytokine IL-1β [175]. Collectively, peripheral oxidative stress and inflammation serve as key factors in determining the effects of PM_2.5_ on the brain.

### 4.3. Particle Exposure and Neurological Disorders

PM_2.5_ exposure has been increasingly linked to a range of neurological disorders, including neurodegenerative and psychiatric conditions. Epidemiological studies that have examined the association between PM_2.5_ or its components and neurological diseases are listed in Table 2. Nunez et al. found that a one standard deviation increase in the organic matter or nitrate concentration was associated with a 6% increase in the annual Parkinson’s disease first hospitalization rate in the multi-pollutant model [176]. Li et al. evaluated concentration–response relationships between each component of PM_2.5_, such as BC, nitrate, organic matter, sulfate, soil particles, and sea salt, and dementia from single-pollutant models and showed that a strong near-linear relationship was observed with BC and soil particles, with no indication of a threshold [177]. Several reports that have examined the effects of organic substances such as PAHs or bioaerosols; however, the majority of these studies have examined the relationship between PM_2.5_ or its inorganic substances in the atmosphere, such as sulfur dioxide, nitrogen dioxide, and ozone, and neurological diseases. Chiang et al. examined statistically significant associations between air pollution exploratory factors and the average number of visits for epileptic seizures per day by month; six air pollutants (i.e., methane, nitric oxide, carbon monoxide, nitrogen dioxide, PM_2.5_, and non-methane hydrocarbons) had positive correlations with the number of visits [178]. As mentioned above, inorganic ions are unlikely to reach the brain even if inhaled; therefore, when considering the association between inorganic ions and neurological disorders observed in these epidemiological studies, secondary neurological effects must be considered rather than transport of PM_2.5_ components to the brain. On the other hand, particulate matter such as BC can reach the brain; thus, interactions with neurological disorders may be a direct effect of the components. When considering the impact of PM_2.5_ components on neurological diseases, it is necessary to consider both toxicological and epidemiological studies; however, it is expected that the knowledge summarized here regarding the pharmacokinetics of PM_2.5_ components will be useful.

Several epidemiological studies examined the interaction of PM_2.5_ and ozone (Table 2). Ozone and PM_2.5_ are interconnected through atmospheric oxidation processes. Ozone can influence the formation and transformation of PM_2.5_ by participating in chemical reactions that lead to secondary aerosol formation. Conversely, PM_2.5_ can affect ozone levels by altering the atmospheric conditions that facilitate ozone production [179,180]. The chemical coupling between ozone and PM_2.5_ involves gas-to-particle conversion processes, where organic and nitrogen oxide emissions (i.e., precursors to ozone) also contribute to PM_2.5_ formation. Interactions between PM_2.5_ components and other environmental factors, or between PM_2.5_ components themselves, are closely related to human health, and the effects of such interactions on transport of components to the brain and expression of neurotoxicity are issues that require further investigation.

**Table 2:** Summary of relevant studies.

Endpoints	Pollutant	Year of Study	Location	Sample Sizes	Reference
				(No. of Cases or Participants)	
Migraine, Headache	PM_2.5_, PM_10_, SO_2_, NO_2_, O_3_, CO	1992–2002	Canada	56,241 ^a^	[181]
				48,022 ^a^	
Migraine	PM_2.5_, PM_10_, SO_2_, NO_2_, O_3_, CO	2006–2011	Taiwan	1,000,000 ^b^	[182]
Ischemic stroke	PM_2.5_	2003–2008	Canada	9,202 ^a^	[183]
Ischemic stroke	PM_2.5_, SO_2_, NO_2_, O_3_, CO	2006–2010	Taiwan	40,009 ^a^	[184]
Ischemic stroke	PM_2.5_, O_3_	2000–2012	USA	2948 ^a^	[185]
Ischemic stroke	PM_2.5_	2007–2015	USA	31,414 ^b^	[186]
Ischemic stroke	PM_2.5_, SO_2_, NO_2_, O_3_, CO	2014–2016	China	2,032,667 ^a^	[187]
Ischemic stroke	total hydrocarbons,	2000–2013	Taiwan	283,666 ^a^	[188]
	nonmethane hydrocarbons				
	PM_2.5_, PM_10_, SO_2_, NO_2_, O_3_, CO,				
	CO_2_, NO_X_, NO, CH_4_				
Ischemic stroke	PM_2.5_	2006–2013	Taiwan	10,035 ^a^	[189]
Ischemic stroke	PM_2.5_	2015–2017	China	155,616 ^a^	[190]
Hemorrhagic stroke	PM_2.5_	2010–2015	Portugal	308 ^a^	[191]
Hemorrhagic stroke	PM_2.5_	2002–2013	Republic of Korea	62,676 ^b^	[192]
Hemorrhagic stroke	PM_2.5_, SO_2_, NO_2_	2012–2014	China	6412 ^a^	[193]
Hemorrhagic stroke	PM_2.5_, PM10, SO_2_, NO_2_, O_3_, CO	2017–2018	Republic of Korea	92 ^a^	[194]
Ischemic, Hemorrhagic stroke	PM_2.5_ SO_4_^2−^, NO_3_^−^, OC, EC	2004–2008	Taiwan	12,982 ^a^	[195]
				3362 ^a^	
Ischemic, Hemorrhagic stroke	PM_2.5_, PM_10_, PM_2.5abs_	2000–2012	Germany	4433 ^b^	[196]
Ischemic, Hemorrhagic stroke	PM_1_, PM_2.5_, PM_10_, OC, EC,	2007–2011	China	9066 ^a^	[197]
	SO_2_, NO_2_, NH_4_^+^ SO_4_^2^−^^, NO_3^−^_,				
	Na^+^, Cl^−^				
Ischemic, Hemorrhagic stroke	PM_2.5_, PM_10_, NO, NO_2_, NO_X_, O_3_	2005–2012	United Kingdom	1800 ^a^	[198]
Ischemic, Hemorrhagic stroke	PM_2.5_	2013–2015	China	1356 ^a^	[199]
Ischemic, Hemorrhagic stroke	PM_2.5_, PM_10_, SO_2_, NO_2_	2013–2015	China	84,535 ^a^	[200]
Ischemic, Hemorrhagic stroke	PM_2.5_, PM_10_, SO_2_, NO_2_, O_3_, CO	2016–2017	China	1063 ^a^	[201]
Ischemic, Hemorrhagic stroke	PM_2.5_, PM_2.5abs_, NO_2_, NO_X_	1996–2012	Australia	1778 ^a^	[202]
Ischemic, Hemorrhagic stroke	PM_2.5_	2014–2018	Israel	74,052 ^a^	[203]
Ischemic, Hemorrhagic stroke	PM_2.5_, EC, NO_2_	2005–2017	Denmark	94,256 ^a^	[204]
PD	PM_2.5_	1999–2010	USA	6,982,678 ^b^	[205]
PD	PM_2.5_, PM_10_	1990–2008	USA	508 ^a^	[206]
PD	PM_2.5_, O_3_	1993–2010	USA	301 ^a^	[207]
PD	PM_2.5_, PM_10_, NO_2_	1995–2006	USA	1556 ^a^	[208]
PD	PM_2.5_, NO_2_, O_3_	2001–2013	Canada	38,475 ^a^	[209]
PD	PM_2.5_, PM_10_, SO_2_, NO_2_, O_3_, CO	2002–2015	Republic of Korea	338 ^a^	[210]
PD	PM_2.5_, BC, organic matter,	2000–2014	USA	197,545 ^a^	[176]
	NO_3_^−^, SO_4_^2−^, sea salt, soil particle				
PD	PM_2.5_	2006–2013	Taiwan	137 ^a^	[211]
PD	PM_2.5_, NO_2_	2009–2018	USA	163 ^a^	[212]
PD	PM_2.5_, NO_2_	1998–2015	USA	346 ^a^	[213]
Dementia	PM_2.5_, PM_10_, NO_2_, O_3_	2001–2009	Spain	1175 ^a^	[214]
Dementia	PM_2.5_, NO_X_	2001–2013	Sweden	364 ^a^	[215]
Dementia	PM_2.5_	2001–2013	Sweden	2253 ^b^	[216]
Dementia	PM_2.5_, NO_2_	2008–2012	USA	398 ^a^	[217]
Dementia	PM_2.5_, PM_coarse_, PM_2.5abs_, NO_2_	2006–2018	United Kingdom	1394 ^a^	[218]
Dementia	PM_2.5_, PM_10_, PM_2.5abs_, NO_2,_ NO_X_	2010–2018	Netherlands	545 ^a^	[219]
Dementia	PM_2.5_, black carbon, organic matter,	2000–2018	USA	309,842 ^a^	[177]
	NO_3_^−^, SO_4_^2−^, NH_4_^+^, soil dust				
Dementia	PM_2.5_	1998–2016	USA	4105 ^a^	[220]
Dementia	PM_2.5_	2006–2019	USA	80,993 ^a^	[221]
Dementia	PM_2.5_, NO_2,_ O_3_	2007–2013	USA	1011 ^a^	[222]
Dementia, AD	PM_2.5_	1993–2010	Sweden	302 ^a^	[223]
Dementia, AD	PM_2.5_, NO_2_	2000–2012	Canada	251,641 ^a^	[224]
Dementia, AD	PM_2.5_	1994–2018	USA	1136 ^a^	[225]
Dementia, AD	PM_2.5_, black carbon, organic matter,	2000–2017	USA	~5.8 million ^a^	[226]
	NO_3_^−^, SO_4_^2−^, NH_4_^+^, soil dust			~2.8 million ^a^	
Dementia, AD	PM_2.5_, NO_2_, O_3_	2000–2018	USA	2,025,130 ^a^	[227]
				804,668 ^a^	
AD	PM_2.5_, PM_10_, SO_2_, NO_2_, O_3_, CO	2001–2010	Taiwan	1399 ^a^	[228]
AD	PM_2.5_	2008–2013	Taiwan	3803 ^a^	[229]
AD	PM_2.5_	1996–2010	USA	998 ^b^	[230]
AD	PM_2.5_	1994–2018	USA	832 ^a^	[231]
AD	PM_2.5_	2018–2020	China	1545 ^b^	[232]
AD	PM_2.5_, PM_10_, SO_2_, NO_2_, O_3_, CO	2010–2014	USA	57,990 ^a^	[233]
Cognitive decline	PM_2.5_, PM_10_, SO_2_, NO_2_, O_3_, CO	2007–2018	Republic of Korea	398,889 ^b^	[234]
Mild cognitive impairment	PM_2.5_, PM_10_, SO_2_, O_3_	2015–2018	China	782 ^a^	[235]
Dementia, AD, vascular dementia	PM_2.5_, black carbon, organic matter,	2006–2021	United Kingdom	5768 ^a^	[236]
	NO_3_^−^, SO_4_^2−^, NH_4_^+^			1860 ^a^	
				1071 ^a^	
AD, non-AD dementia, PD	PM_2.5_	2007–2014	USA	1503 ^a^	[237]
				4955 ^a^	
				570 ^a^	
Brain tumors	PM_2.5_, PM_10_, NO_2,_ NO_x_	1993–2013	Denmark	121 ^a^	[238]
Brain tumors	UFP (<0.1 µm), PM_2.5_, PM_10_	1991–2016	Canada	1400 ^a^	[239]
Epilepsy	PM_2.5_, SO_2_, NO_2_, O_3_	2013–2014	China	20,368 ^a^	[240]
Epilepsy	PM_2.5_, PM_10_, SO_2_, NO_2_, O_3_, CO, NO,	2009–2013	Taiwan	108,175 ^a^	[178]
	CH_4_, non-methane hydrocarbons				
Multiple sclerosis	PM_2.5_, PM_10_, SO_X_, NO_X_, NH_3_, CO	1990–2015	Italy	927 ^a^	[241]
Multiple sclerosis	PM_2.5_	1998–2018	Italy	683 ^a^	[242]
Multiple sclerosis	PM_2.5_	2017	195 countries	1,761,078 ^a^	[243]
Multiple sclerosis	PM_2.5_, PM_10_	2013–2022	Thailand	126 ^a^	[244]
Schizophrenia	PM_2.5_	2010–2015	USA, China	46 ^a^	[245]
Depression	PM_2.5_, SO_2_, NO_2_, NO_X_, O_3_, CO,	2010–2115	Poland	318,779 ^a^	[246]
	benzo(a)pyrene, Pb				
Sleep disorder	PM_1_, PM_2.5_, PM_10_, SO_2_, NO_2_,	2012–2013	China	2,304 ^a^	[247]
	O_3_, CO				
Sleep disorder	PM_2.5_	2012–2015	USA	51,562 ^b^	[248]
Sleep disorder	PM_2.5_, PM_10_, SO_2_, NO_2_, O_3_	2018–2019	China	87,734 ^a^	[249]
Hearing loss	PM_2.5_, PM_10_, SO_2_, NO_2_, O_3_	2015	Republic of Korea	817 ^a^	[250]
Hearing loss	PM_2.5_, SO_2_, NO_2_ O_3_, CO	2011–2019	Taiwan	850 ^a^	[251]
Olfactory decline	PM_2.5_, PM_10_	2016–2017	Mexico	120 ^b^	[252]
Olfactory decline	PM_2.5_	2001–2016	Sweden	1774 ^b^	[253]
Anosmia	PM_2.5_	2013–2016	USA	538 ^a^	[254]
Amyotrophic Lateral Sclerosis	PM_2.5_	1989–2013	Denmark	3983 ^a^	[255]
Developmental disorders	PM_2.5_, SO_2_, NO_2_, O_3_, CO,	2016–2020	Republic of Korea	843,134 ^b^	[256]
	Pb, Cd, Cr, Cu, Mn, Fe, Ni, As				
Nervous system anomalies	PM_2.5_	2004–2018	Taiwan	12,383 ^a^	[257]
Cerebral palsy	PM_2.5_, NO_2_, O_3_	2002–2017	Canada	3170 ^a^	[258]
Tic disorders	PM_2.5_	2004–2017	Taiwan	5902 ^a^	[259]
Delirium	PM_2.5_, PM_10_, SO_2_, NO_2_, CO	2014–2015	China	559 ^a^	[260]
Brain volume, Covert brain infarcts	PM_2.5_	1998–2001	USA	943 ^b^	[261]
Gray/white matter volumes	PM_2.5_	1996–2006	USA	1403 ^b^	[262]
Cerebral vascular resistance	PM_2.5_	2005–2008	USA	482 ^b^	[263]

^a^: cases, ^b^: participants. PD, Parkinson’s disease; PM_2.5abs_, PM_2.5_ absorbance is a measure of soot content; PM_coarse_, particulate matter with aerodynamic diameter between 2.5 and 10 µm; SO_X_, sulfur oxides; NO_X_, nitrogen oxides; UFP, ultrafine particles.

## 5. Conclusions

Effects of PM_2.5_ on the human respiratory and circulatory systems have become clear over recent decades. In particular, the respiratory system, including the bronchi and lungs, is directly affected by inhaled PM_2.5_; thus, the respiratory system is the target of all chemical components of in PM_2.5_. Epidemiological studies are beginning to show that the brain and nervous system are targets of PM_2.5_. However, the pathway in which PM_2.5_ affects the brain is not well understood. There are several neurological disorders for which treatment is limited or cannot be expected to be cured. In addition, the brain has poor metabolic and excretory functions, and particulate matter or chemicals that enter the brain can act for a long time in the brain. Therefore, suppressing the neurological effects of PM_2.5_ is very important from a public health perspective. Reducing PM_2.5_ emissions is a simple and most effective method. However, ceasing economic activity is not realistic, and since PM_2.5_ is also released from nature, it is not possible to reduce the PM_2.5_ concentration in the air to zero. PM_2.5_ is a complex of many different chemicals; however, as mentioned in this review, it is believed that only a limited number of chemicals have a direct effect on the brain. Focusing on the pharmacokinetics of each component is considered an essential process for understanding the neurological effects of PM_2.5_. Clarification of the origin of each component in PM_2.5_ and its effect on the CNS will enable measures to be taken against the emission source of each component, and the onset and exacerbation of nervous system diseases caused by PM_2.5_ can be greatly suppressed. In this review, we have summarized the pathways by which each component of PM_2.5_ reaches the brain and its effects on the CNS. We believe that this review will be useful in maintaining human health through elucidating the neurotoxicity mechanism of inhaled PM_2.5_ and understanding the onset and progression of neurological disorders related to air pollution.

## Figures and Tables

**Table 1:** Summary of action mechanisms of major PM_2.5_ components in the brain.

Classification	Components	Action	Reference
Water-soluble ions	Sulfur oxides	(Do not affect the brain)	
	Nitrogen oxides	(Do not affect the brain)	
Metals	Fe	ROS generation	[144]
		Ferroptosis	[145]
	Zn	Excitotoxicity	[146]
		Mitochondrial dysfunction	[147]
		Oxidative stress	[148]
		Neuroinflammation	[148]
	Cr	Neurotransmitter disruption	[149]
		Oxidative stress	[150]
	Cu	BBB disruption	[151]
		Mitochondrial dysfunction	[152]
		Oxidative stress	[153]
	Mn	Impaired neurotransmission	[154]
		Oxidative stress	[155]
		Neuroinflammation	[155]
Carbons	Black carbon	ER stress	[156]
		Oxidative stress	[156]
PAHs	Benzo[a]pyrene	Synaptic dysfunction	[157]
		Neuroinflammation	[158]
		Oxidative stress	[159]
		DNA methylation	[160]
		Tumorigenesis	[161]
	Benzo[k]fluoranthene	Oxidative stress	[160]
		DNA methylation	[160]
Quinones	1,2-naphthoquinone	(No literature)	
	9,10-phenanthrenequinone	(No literature)	
	6PPDQ	Oxidative stress	[162]
		Neuroinflammation	[162]
		Apoptosis	[162]
Plastics	MPs/NPs	Neuroinflammation	[163]
		Oxidative stress	[163]
		Mitochondrial dysfunction	[164]
		Neurotransmitter disruption	[165]
Bioaerosols	Bacteria	BBB disruption	[166]
	LPS	BBB disruption	[167]
	Mold	Innate immune activation	[168]
